# A Novel Blunge Calibration Intelligent Feature Classification Model for the Prediction of Hypothyroid Disease

**DOI:** 10.3390/s23031128

**Published:** 2023-01-18

**Authors:** Munisamy Shyamala Devi, Venkatesan Dhilip Kumar, Adrian Brezulianu, Oana Geman, Muhammad Arif

**Affiliations:** 1 Department of CSE, Vel Tech Rangarajan Dr. Sagunthala R&D Institute of Science and Technology, Chennai 600062, India; 2 Faculty of Electronics, Telecommunications and Information Technology, “Gheorghe Asachi” Tehnical University, 700506 Iasi, Romania; 3 Greensoft Ltd., 700137 Iasi, Romania; 4 The Computers, Electronics and Automation Department, Faculty of Electrical Engineering and Computer Science, “Stefan cel Mare” University of Suceava, 720229 Suceava, Romania; 5 Department of Computer Science, Superior University, Lahore 54000, Pakistan

**Keywords:** machine learning, regression, outlier, MAE, MSE, EVS

## Abstract

According to the Indian health line report, 12% of the population suffer from abnormal thyroid functioning. The major challenge in this disease is that the existence of hypothyroid may not propagate any noticeable symptoms in its early stages. However, delayed treatment of this disease may lead to several other health problems, such as fertility issues and obesity. Therefore, early treatment is essential for patient survival. The proposed technology could be used for the prediction of hypothyroid disease and its severity during its early stages. Though several classification and regression algorithms are available for the prediction of hypothyroid using clinical information, there exists a gap in knowledge as to whether predicted outcomes may reach a higher accuracy or not. Therefore, the objective of this research is to predict the existence of hypothyroidism with higher accuracy by optimizing the estimator list of the pycaret classifier model. With this overview, a blunge calibration intelligent feature classification model that supports the assessment of the presence of hypothyroidism with high accuracy is proposed. A hypothyroidism dataset containing 3163 patient details with 23 independent and one dependent feature from the University of California Irvine (UCI) machine-learning repository was used for this work. We undertook dataset preprocessing and determined its incomplete values. Exploratory data analysis was performed to analyze all the clinical parameters and the extent to which each feature supports the prediction of hypothyroidism. ANOVA was used to verify the F-statistic values of all attributes that might highly influence the target. Then, hypothyroidism was predicted using various classifier algorithms, and the performance metrics were analyzed. The original dataset was subjected to dimensionality reduction by using regressor and classifier feature-selection algorithms to determine the best subset components for predicting hypothyroidism. The feature-selected subset of the clinical parameters was subjected to various classifier algorithms, and its performance was analyzed. The system was implemented with python in the Spyder editor of Anaconda Navigator IDE. Investigational results show that the Gaussian naive Bayes, AdaBoost classifier, and Ridge classifier maintained the accuracy of 89.5% for the regressor feature-selection methods. The blunge calibration regression model (BCRM) was designed with naive Bayes, AdaBoost, and Ridge as the estimators with accuracy optimization and with soft blending based on the sum of predicted probabilities of classifiers. The proposed BCRM showed 99.5% accuracy in predicting hypothyroidism. The implementation results show that the Kernel SVM, KNeighbor, and Ridge classifier maintained an accuracy of 87.5% for the classifier feature-selection methods. The blunge calibration classifier model (BCCM) was developed with Kernel SVM, KNeighbor, and Ridge as the estimators, with accuracy optimization and with soft blending based on the sum of predicted probabilities of classifiers. The proposed BCCM showed 99.7% accuracy in predicting hypothyroidism. The main contribution of this research is the design of BCCM and BCRM models that were built with accuracy optimization with soft blending based on the sum of predicted probabilities of classifiers. The BCRM and BCCM models uniqueness’s are achieved by updating the estimators list with the effective classifiers and regressors that suit the application at runtime.

## 1. Introduction

For the accurate diagnosis of thyroid illness, functional data from the thyroid gland must be interpreted. Hypothyroidism is a condition where the thyroid gland in the body is unable to secrete thyroid hormone. Women are eight times as likely as males to suffer a thyroid condition. The thyroid condition tends to worsen and persist with ageing and may affect adults differently compared with children. The thyroid gland mainly helps in controlling the body’s metabolism. Globally, thyroid disorders have begun to become more prevalent. For instance, one in eight women in Romania suffers from thyroid cancer. Approximately 30% of Romanians have endemic goiters. A limited diet, the use of drugs, anxiety, sickness, trauma, pollutants, and other elements can all affect thyroid function. Predetermined data sets can be categorized using classification, and this is a crucial supervised learning data-mining approach.

## 2. Literature Review

We examined, using machine learning, the thyroid data included in UC Irvine’s knowledge discovery archive [1]. Thyroid disease has been classified as a common thyroid dysfunction in the general population. Our findings demonstrate the great accuracy of each of the aforementioned classification models, in which the decision tree model has the highest categorization rate. The infrastructure for creating and evaluating the models was provided by the KNIME analytics platform and Weka, which are two data-mining applications [2]. Classification is commonly used in the healthcare sector to inform business choices, diagnose patients, and provide them with exceptional care [3].

The precise estimation of thyroid gland operational information is critical for thyroid diagnosis. The thyroid gland mainly aids in the control of an individual’s metabolism. The types of thyroid disease are determined by the production of either too little or too much thyroid hormone. Various neural networks have been used in this study to aid in the analysis of thyroid disease [4]. These networks aimed to diagnose thyroid disease by using a new hybrid machine-learning method that includes our classification system. A method for solving this diagnosis problem via classification was obtained by hybridizing AIRS with an advanced fuzzy weighted pre-processing. A cross-validation analysis was used to determine the technique’s soundness for sampling variability [5]. A novel hybrid machine learning approach that incorporates this classification system was used to identify thyroid illness. AIRS and sophisticated fuzzy weighted pre-processing were combined to create a strategy for categorizing this diagnostic issue. By using cross-validation analysis, the technique’s robustness for sampling variability was evaluated [6]. The expansion of scientific knowledge and the massive production of data have resulted in an exponential growth in databases and repositories. One of these rich data domains is the biomedical domain. A large amount of biomedical data is available, ranging from clinical symptom details to various types of biochemical data and imaging device outputs. Mechanically retrieving biological information from images and reshaping them into machine-readable knowledge is a challenging task, because the biomedical domain is vast, dynamic, and complicated. Data mining can improve the quality of biomedical pattern extraction [7].

A backpropagation algorithm is an early method for the detection of thyroid disease. An advanced neural network (ANN) was created using backpropagation of error for prior disease diagnosis. Afterward, this ANN was trained using empirical values, and testing was performed using information that had not been used during the training process [8]. Data collection is an important methodological approach in the field of medical disciplines, because efficient techniques for analyzing and identifying disorders are required. Data mining applications are used in clinical governance, health information technology, and patient care systems. It is also important in determining disease resilience. The popular data mining techniques used to recognize the complex parameters of the nutrition data set are classification and clustering [9]. 

A novel approach was used for the detection of three types of anomalous red blood cells, known as poikilocytes, that were found in iron-deficient blood smears. The classification and counting of poikilocytes are critical steps in the rapid recognition of iron deficiency anemia disease. The three basic poikilocytes in IDA are dacrocyte, elliptocyte, and schistocyte [10]. High-dimensional biomedical datasets contain thousands of features that are used to diagnose genetic diseases, but their predictive accuracy is affected by numerous irrelevant or weak connection features. While minimizing computation complexity in data mining, feature-selection techniques enable classification models to precisely discover patterns in features and determine a feature vector from an initial set of features. An enhanced shuffled frog-leaping algorithm (ISFLA) is presented in this paper, and it explores the space for potential subsets to choose the set of attributes that maximizes prognostication while minimizing irrelevant attributes in high-dimensional biological data [11]. The latest ANN-based finite impulse response extreme learning machine (FIR-ELM) was used to further analyze the categorization of two binary bioinformatics datasets into leukemia and colon tumor. To investigate the hidden layer of the neural classifier’s FIR-ELM for the smoothing capabilities of feature identification, we performed a time series analysis of the microarray samples. Afterward, we determined how linearly divergent the data patterns in the microarray datasets were [12]. 

The optimal feature-selection problem, and its authors, describe a coherent analytical foundation that can retrofit successful heuristic criteria, indicating the approximate solutions made by each method [13]. The outcome of a microstructure heart arrhythmia detection system based on electrocardiography, ECG, and signal features was analyzed. These signals came from the MIT/BIH arrhythmia directory. Initially, Hermitian basis functions were used to model the ECG beats. The width parameter—sigma—of HBF was optimized in this step to minimize model error. The extracted features, which contain the model’s boundary conditions, were used as input for the k-nearest neighbor classifier, KNN, to evaluate the model’s effectiveness [14]. Approximately 90% of patients with Parkinson’s disease are predicted to have vocal and speech issues. Vocal folds are often weakened by this infection, causing the patients to have an unnatural voice. In the present study, different samples from the auditory signal of patients with Parkinson’s disease and healthy individuals were gathered. The data classification was then completed using the KNN classification approach based on varied amounts of optimized features after the optimized features that influenced the data classification process were determined using a genetic algorithm [15]. Although thrombolysis reduces impairment and increases survival rates in patients with ischemic stroke, some people continue to suffer detrimental effects. Consequently, it will be beneficial, when making health decisions, to predict how patients with myocardial infarction might react to regional rehabilitation [16].

Straightforward, mathematical assessment criteria need to be established to generate and quantify pragmatic forecasts in cerebral ischemia with data that are readily available post-surgery in the emergency unit. Regression was used to investigate the causes of inferior outcomes in the originating sample of formerly independent people with information systems. The covariate correlations from the computed holistic framework were used to build a scoring model based on integers for each correlation coefficient, and the average of the scores for the criterion was used to obtain the total result [17]. This process aims to offer a self-contained method for improving learning-argumentation frameworks that employ deformation key frames of MR images to aid in the rational frameworks of ischemic stroke diagnosis. Anthropological, physiological, and statistical approaches were gathered from the fragmented tumors to form a feature set that was then further defined using classification techniques. The results of the recommended approach, which accurately designates electromagnetic fields as vascular tumors with a 93.4% accuracy, are significantly superior to those of the classification model [18]. Among many other clinical and imaging parameters, ageing and the severity of a hemorrhage are immediate, precise indicators of the likelihood of SICH and the results of treatment following intravenous infusion therapy [19]. The use of aided technology for stroke could reduce the evaluation period, improve prediction performance, make it simpler to discriminate between different types of ICH, and reduce the chance of human error [20]. 

One study presents the improvements in learning methods and developments that are in line with the different varieties and manifestations of dyslexia. This study opens with a discussion of cosmic mythology and examines how learning environments that consider student’s skills and requirements can be combined with the appropriate assistive technology to deliver effective e-learning experiences and reliable instructional resources. The Ontology Web Language, a data-handling framework that enables programmers to handle both the substance and the introduction of the data available on the web, was used to generate the metaphysics used in this evaluation [21]. The methodology was designed and implemented to help identify the fundamental problems that may affect students learning to read or write and problems that may then lead to further problems with memory cognition. This strategy was used to assist activists and parents in understanding the issue of dyslexia and to put children on the right path to academic success [22]. Participants, with and without dyslexia, used an online game with language-independent melodic and visual components to communicate in different languages. A total of 178 participants were involved. The analysis revealed nine game measures for Spanish children with and without dyslexia that had significant differences and which could be used in current projects as a justification for speech independent exploration [23]. Quantitative and artificial intelligence-based methods are recommended to instinctually seek innovative and complicated features that consider reliable credentials among dyslexic and control listeners and to support the hypothesis that the majority of differences between dyslexic and talented readers are located on the left side of the brain. Unexpectedly, these devices have also demonstrated how high pass signals carry vital information [24]. Their analysis revealed certain remarkable EEG patterns associated with autism, which is a learning disability with a neurological basis. Although EEG signals contain important information about mental processes, understanding these practices is typically indirect because of their intricate nature. This approach identifies the optimal EEG terminals and brain regions for order and the extraordinary EEG signals produced during writing and composition in adults with dyslexia [25]. The central idea is to begin creating code language for scripting matrices by using the Boolean algebra features of the codes and to present two decryption techniques that enable the identification and retrieval of potential faults or rejection [26]. Dynamic subsamples of ocean climate predictions of surface temperature anomalous outliers in the Tasman Sea were enhanced by the employment of reports of extreme sea-surface temperature that derived from the space station’s geographical position system. The parameters of an extreme value distribution were predicted using regression analysis on the important marine meteorological data in a probabilistic conceptual structure [27]. Additional or standardized nuclear approaches can be employed to overcome the constraints of current investigations into the original sources of seafood. Cross luminescence and carbon isotope analysis have been used to pinpoint the production method and geographic origin of Asian freshwater fish [28]. 

Security concerns that develop during earthquake activity and during periods when the threat of earthquake activity is at its peak, should always be handled probabilistically [29]. For this study, the two quantifiable methods for estimating the likelihood of seismic behavior to affect important and relatively low- and mid-rise structures are presented. The non-linear and linear systems separately and simultaneously assess the injury concerns of an inclined plane exposed to uncontrollable shaking and atmospheric threats, respectively. These systems are divided into three parts: danger showcase; underpinning delicacy examination; and destruction likelihood processing [30]. 

Numerous well-known classification methods, such as decision tree, ANN, logistic regression, and naive Bayes were examined for one study. Then, bagging and boosting procedures were created to increase the durability of these frameworks. Additionally, random forest was considered when the investigation was evaluated. The best result of the sickness risk random-forest strategy was employed for classification according to outcomes. Subsequently, a web application for predicting future occurrences was created using this approach. People with higher chance of getting diabetes were included in the diabetes risk class [31]. Heart rate variation information derived from ECG signal data were used for a further investigation. Here, when CNN-LSTM was originally tested with the HRV data, the prediction accuracy was 90.9%. By using CNN-LSTM integration, the accuracy was improved to 95.1%, and by using five-fold cross-validation based on the same data, the efficiency was enhanced to 93.6%. The cross-validation efficiency is the maximum priority currently available for the automatic identification of hypertension [32]. The information was subjected to several machine-learning approaches, and categorization was carried out using a range of strategies, in which regression analysis resulted in the highest accuracy of 96%. With a 98.8% accuracy rate, the AdaBoost classifier was the pipeline’s most appropriate prediction. Two independent datasets were used to compare the accuracy of the machine-learning methods. The algorithm clearly enhanced the diabetes prediction accuracy and precision when utilizing this information compared to previous resources [33]. 

Additionally, the mellitus dataset was used to evaluate the effectiveness of various suggested deep neural networks and machine learning classification techniques. The other methods had an accuracy that is higher than 90%; for instance, the XGBoost classifier achieved a performance of approximately 100.0% [34]. Both cutting-edge methodologies and some well-known machine learning strategies were contrasted with the DNN algorithm. The suggested technique, which is dependent on the DNN technique, delivered impressive outcomes, with an accuracy of 99.75% and an F1-score of 99.66% [35]. Some papers have been published by authors that report the application of SVM, KNN, or other ML tools in biomedical applications [36,37,38,39,40]. Automated medical diagnostic systems can be easily accessed by the general public, especially by those who cannot afford quality medical care. This methodology essentially combines soft and harsh inputs. A wide range of typical symptoms, including fever, headaches, and cough, were considered soft inputs. Each chosen illness was associated with a range of universal symptoms. Images of the tongue were considered hard inputs because doctors frequently utilize them to identify a variety of illnesses. Hard input analysis was split into two stages: chromatic color analysis and statistical analysis based on texture. After being decoded from the hard and soft inputs, the feature vectors were supplied to a neural network to create a classification mode [41].

## 3. Research Methodology

A hypothyroidism dataset from the UCI containing 3163 patient details with 23 independent features and one dependent feature (https://archive.ics.uci.edu/ml/datasets/thyroid+disease, accessed on 12 January 2023) was used as shown in Equation (1).

(1) HY={[H1,H2,H3,……….., H23], [D]}

where HY represents the hypothyroid dataset. 

We undertook dataset preprocessing and determined its incomplete values. The incomplete data were computed for the hypothyroidism dataset by computing the mean of input values for each attribute with Equation (2).

(2) $$HY_{ij}=\frac{1}{23}\;\sum_{i=1}^{23}\;\sum_{j=1}^{3163}\;\sum_{v=1}^{23}{\left(HY_{ij}\right)}^{v}$$

Equation (2) expresses the estimation of the null data information and attribute scaling of the vehicle motion dataset with Equation (3).

(3) $$HY^{\prime }=\frac{1}{23}\sum_{V=1}^{23}\;{\left(H{Y^{\prime }}_{HY}\right)}^{V}$$

where $HY^{\prime }$ is the complete processed dataset without null values. The imputation deviation of features was measured using the average of the estimated variance within the hypothyroidism dataset as shown in Equation (4).

(4) $$HY^{\prime }=\frac{1}{23}\sum_{V=1}^{23}{\left(H{Y^{\prime }}_{HY}\right)}^{V}=\frac{1}{23}\sum_{v=1}^{23}\left(variance{\left({HY^{\prime }}_{HY}\right)}^{v}\right)$$

The imputed dataset was estimated with the interval value “Interval”of each feature by finding its variance and was estimated using Equation (5).

(5) $$Interval=HY^{\prime }-\frac{1}{v-1}\sum_{v=1}^{7}HY^{\prime }-\sum_{v=1}^{23}(variance{\left(H{Y^{\prime }}_{HY}\right)}^{v}\;$$

**Figure 1:**
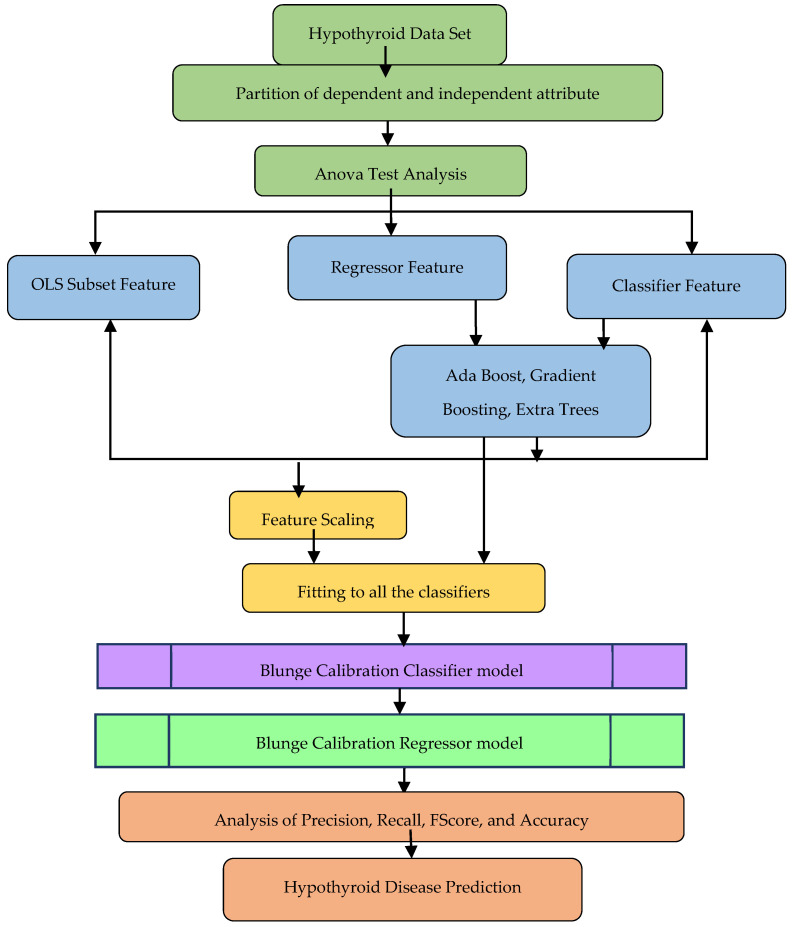
Proposed system workflow.

The overall architecture of the work is shown in Figure 1. The following contributions are provided in this work. 

The complete processed data including incomplete values that contained the complete variance were estimated using Equation (6) as follows:

(6) $$FinalHY=HY^{\prime }+\left(\frac{v+1}{v}\right)\times Interval$$

An exploratory data analysis was performed to analyze all the clinical parameters and the extent to which each feature supports the prediction of hypothyroidism. The number of parameters, the correlation of all variables, as in the following equation, and the data type of the characteristics as given in Equation (7), were evaluated by subjecting a dataset to exploratory prescriptive data analysis.

(7) $$corr=\left[\frac{\sum_{h=1}^{123}\left(HY_{h}-\;\underline{H}Y\right)\;\sum_{d=1}^{1}\left(D_{d}-\;\underline{D}\right)\;}{\sqrt{\sum_{h=1}^{18}{\left(HY_{h}-\;\underline{H}Y\right)}^{2}}\sqrt{\sum_{d=1}^{1}{\left(D_{d}-\;\underline{D}\right)}^{2}\;}}\right]$$

As stated in Equations (8)–(10), the dataset was divided into training and testing data with an 80:20 ratio. Python script was used for the implementation by using the Spyder platform and Anaconda navigator.

(8) $$Train\left(\underline{HY}\right)=\frac{80\;percent\;of\;{\left(Rand\right)}^{2}}{hy}\frac{\left(HY-hy\right)}{HY}$$

(9) $$Test\left(\underline{HY}\right)=\frac{20\;percent\;of\;{\left(Rand\right)}^{2}}{hy}\frac{\left(HY-hy\right)}{HY}$$

(10) $${\left(Rand\right)}^{2}=\left[\frac{\;\sum_{h=1}^{23}{\left(HY_{h}-HY_{h}\right)}^{2}}{HY_{h}-1}\right]$$

**Figure 2:**
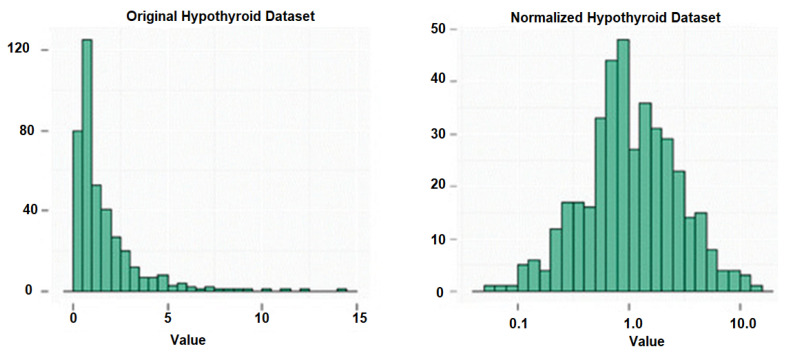
Normalization of the hypothyroidism dataset.

ANOVA test was carried out to verify the F-statistic values of all features with a PR(>F) <0.05 that highly influence the target. Then, hypothyroidism was predicted using various classifier algorithms, and the performance was analyzed. The original dataset was subjected to normalization in order to make it ready for application of the ANOVA test. This is achieved by using the Box–Cox method from the statistical package of NumPY and pandas. The Box–Cox approach transforms and normalizes the data to handle non-normally distributed data. The results obtained from the Box-Cox method is shown below in Figure 2.

**Figure 3:**
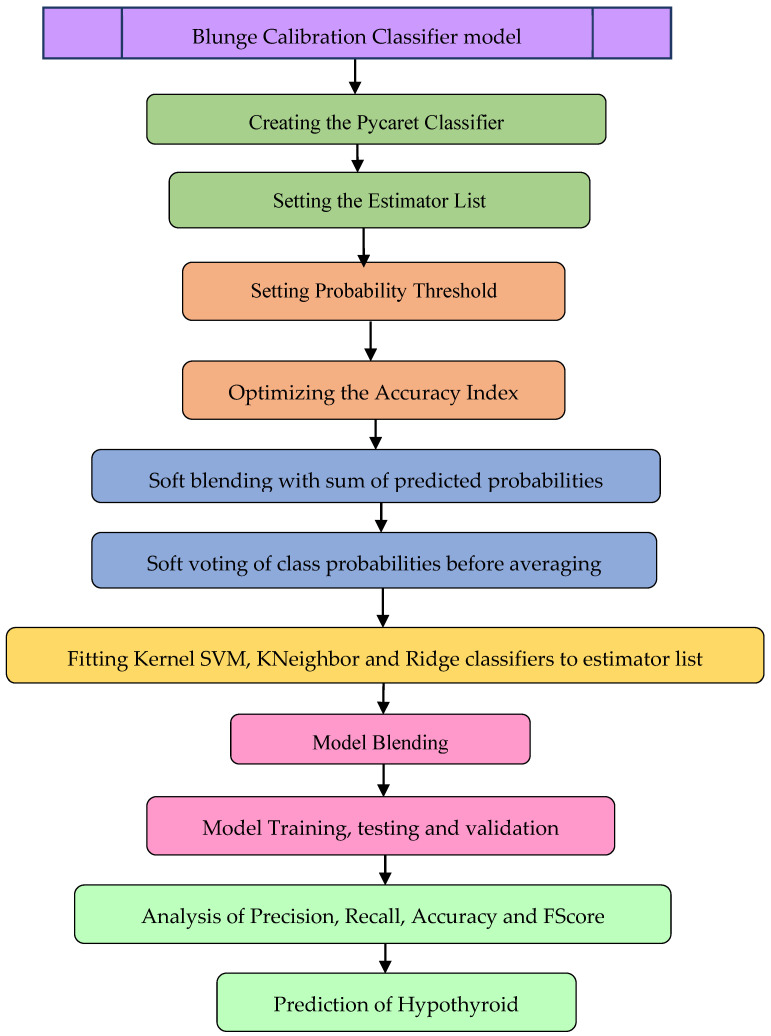
Blunge calibration classifier model workflow.

The original dataset was subjected to dimensionality reduction using the regressor and classifier feature-selection algorithms to determine the best subset components for predicting hypothyroidism. The feature-selected subset of the clinical parameters was subjected to various classifier algorithms, and the performance was analyzed using the specified metrics. The implementation was carried out with python in Spyder editor with Anaconda Navigator IDE. Investigational results show that the Gaussian naive Bayes, AdaBoost classifier, and Ridge classifier maintained an accuracy of 89.5% for the regressor feature selection methods. The blunge calibration regression model, as shown in Figure 3, was created with naive Bayes, Ada boost, and Ridge as the estimators with accuracy optimization using soft blending based on the sum of predicted probabilities of classifiers as shown in Equations (11)–(15).

(11) $$GuassianNB=\frac{1}{\sqrt{2\pi \sigma_{h}^{2}}}\;exp\left(-\frac{{\left(HY_{h}-Mean_{s}\right)}^{2}}{2\sigma_{s}^{2}}\right)$$

(12) $$Adaboost=\frac{\sum_{h=1}^{23}{\left(D_{d}-HY_{h}^{23}\;\hat{\beta }\right)}^{2}}{2}+\lambda \left(\frac{1-\alpha }{2}\;\sum_{h=1}^{23}{\hat{\beta }}_{h}^{2}+\alpha \sum_{h=1}^{23}\left|{\hat{\beta }}_{h}^{2}\right|\right)$$

(13) $$\hat{\beta }=argmin\left[\sum_{s=1}^{9}\left|\left(D_{d}\right)-\sum_{h=1}^{23}\left(HY_{h}\right)\right|\right]$$

(14) $$Ridge=\lambda \left(\frac{1-\alpha }{2}\;\sum_{h=1}^{23}{\hat{\beta }}_{h}^{2}+\alpha \sum_{h=1}^{23}\left|{\hat{\beta }}_{h}^{2}\right|\right)$$

(15) BCRM=Estimator{(GuassianNB, Adaboost, Ridge)}

**Figure 4:**
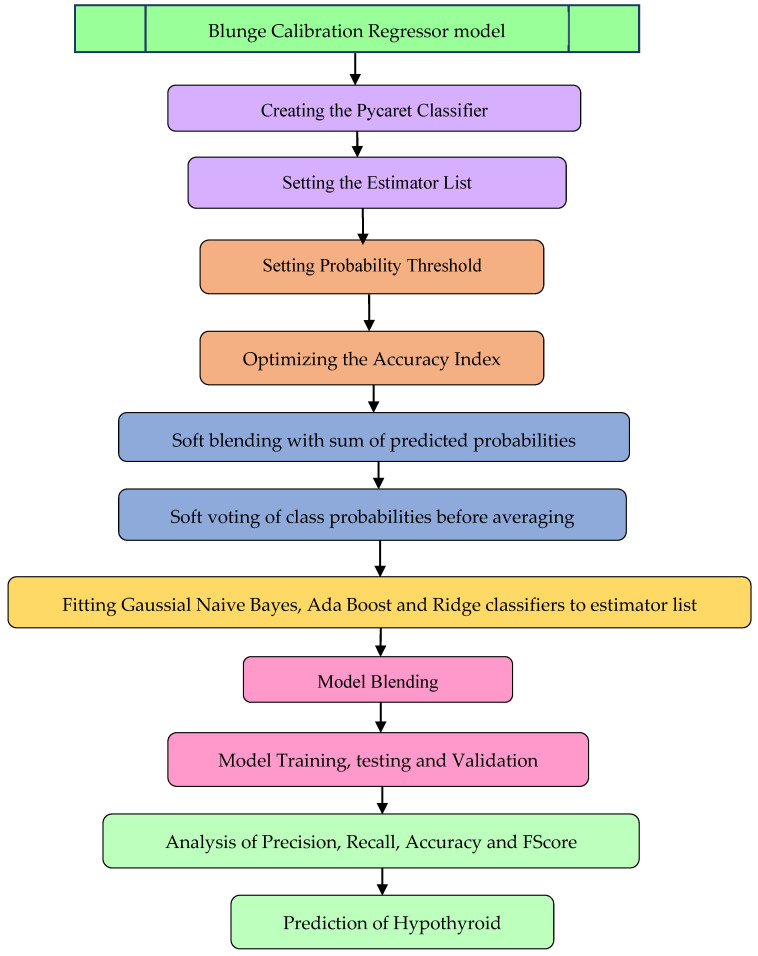
Blunge calibration regressor model workflow.

The implementation results show that the Kernel SVM classifiers, KNeighbor classifier, and Ridge classifier maintained an accuracy of 87.5% for the classifier feature-selection methods. The blunge calibration classifier model, as shown in the Figure 4, was created with Kernel SVM, KNeighbor, and Ridge as the estimators with accuracy optimization using soft blending based on the sum of predicted probabilities of classifiers as shown in Equations (16)–(19).

(16) $$KNN\left(HY,\;D\right)=\sqrt{\sum_{v=1}^{23}{\left(HY_{h}-D_{h}\right)}^{2}}$$

(17) $$kernel\left(HY,\;HY^{\prime }\right)=exponential\left(\frac{{\left[-\|HY-VHY^{\prime }\|\right]}^{2}}{2\sigma^{2}}\right)$$

(18) $$kernelSVM\left(HY\right)=\sum_{v=1}^{7}\propto \times B\times kernel\left(HY,\;HY^{\prime }\right)+vector$$

(19) BCCM=Estimator{(KNN, kernelSVM, Ridge)}

## 4. Implementation Setup

**Figure 5:**
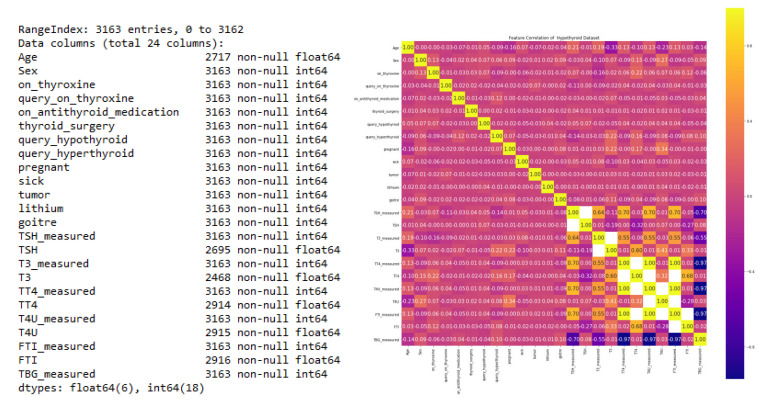
Statistical information and correlation matrix of the dataset.

The hypothyroid dataset with 3163 rows and 24 feature components from UCI was used for data preprocessing. The dataset information is shown in Figure 5.

**Figure 6:**
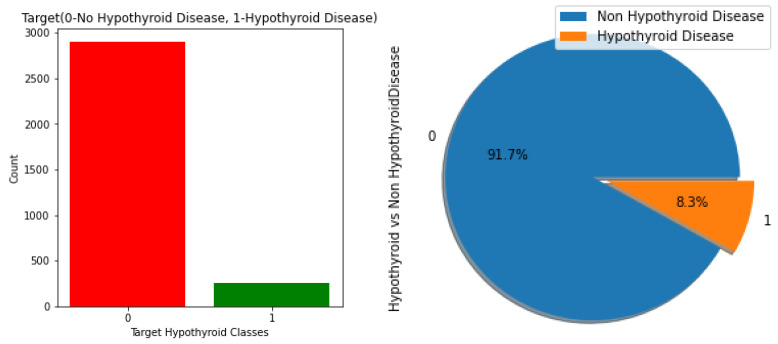
Density plot and target distribution of the hypothyroidism dataset.

Implementation was undertaken with Python under an NVidia Tesla V100 GPU server with 30 training epochs and a batch size of 64. All clinical parameters were analyzed by determining the relationship between each feature and its correlation, as shown in Figure 6.

### 4.1. Anova Test Analysis

ANOVA was carried out to analyze those attributes of the dataset with PR(>F) < 0.05 that highly influence the target. ANOVA was applied to the dataset features, and the results show that the features (thyroid surgery, pregnant, tumor, lithium) have values of PR(>F) > 0.05 and do not contribute to the target, the results are shown in Table 1.

### 4.2. Results and Discussion

Hypothyroidism was predicted using various classifier algorithms before and after feature scaling, the performances were analyzed, and the results are shown in Table 2 and Table 3.

The raw dataset was subjected to dimensionality reduction by using AdaBoost, gradient boosting regressor, extra trees, and random forest regressor feature-selection methods, and the feature importance values of each attribute of the hypothyroidism dataset before and after feature scaling are shown in Table 4 and Table 5. The raw dataset was subjected to dimensionality reduction using AdaBoost, gradient boosting, extra trees, and random forest classifier feature-selection methods, and the feature importance values of each attribute of the hypothyroid dataset before and after scaling are shown in Table 6 and Table 7. 

A feature importance index of all the regressor and classifier feature-selection methods of the hypothyroid dataset, before and after feature scaling, was also compared, and the results are shown in Table 8.

The feature-selected subset of the AdaBoost regressor was applied to the classifiers, and the performance was analyzed. The results are shown in Table 9 and Table 10.

The feature-selected subset of the gradient boosting regressor was applied to the classifiers, the performances before and after feature scaling were analyzed, and the results are shown in Table 11 and Table 12.

The feature-selected subset of extra trees regressor was applied to the classifiers, the performances before and after scaling were analyzed, and the results are shown in Table 13 and Table 14.

The feature-selected subset of random forest regressor was applied to the classifiers, the performances before and after feature scaling were analyzed, and the results are shown in Table 15 and Table 16.

**Figure 7:**
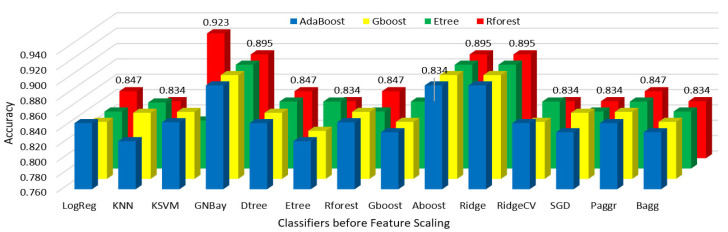
Regressor feature importance performance of all classifiers before scaling.

**Figure 8:**
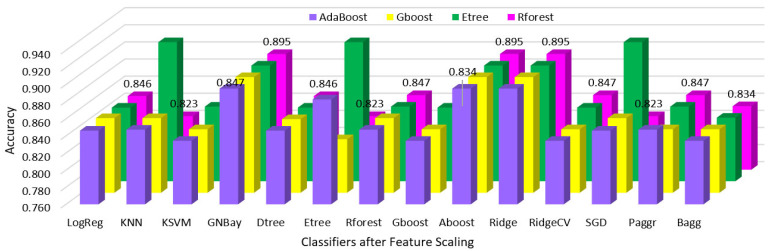
Regressor feature importance performance of all classifiers after scaling.

The performances of all classifiers after reduction with the feature importance of the AdaBoost, gradient boost, extra tree, and random forest regressors before and after feature scaling are shown in Figure 7 and Figure 8.

The feature selected subset of the AdaBoost classifier was applied to the other classifiers, the performances were analyzed, and the results are shown in Table 17 and Table 18.

The feature-selected subset of the gradient boosting classifier was applied to the classifiers, the performances before and after feature scaling were analyzed, and the results are shown in Table 19 and Table 20.

The feature selected subset of the extra trees classifier was applied to the other classifiers, the performances were analyzed, and the results are shown in Table 21 and Table 22.

The feature-selected subset of the random forest classifier was applied to the other classifiers, the performances before and after feature scaling were analyzed, and the results are shown in Table 23 and Table 24.

**Figure 9:**
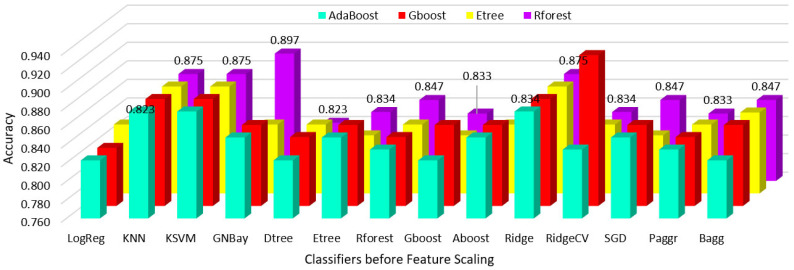
Classifier feature importance performance of all classifiers before scaling.

**Figure 10:**
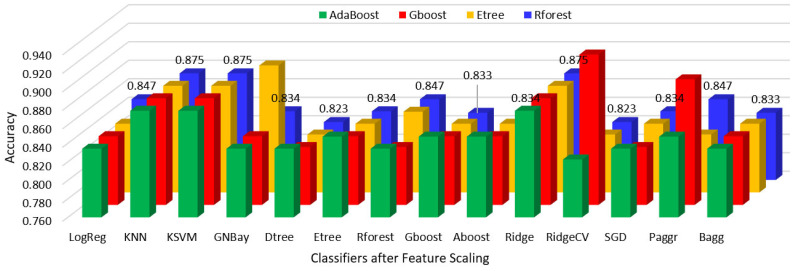
Classifier feature importance performance of all classifiers after scaling.

The performances of all classifiers after reduction with the feature importance of the AdaBoost, gradient boost, extra tree, and random forest classifiers before and after feature scaling are shown in Figure 9 and Figure 10.

The overall dataset was analyzed with the OLS features, such as *p* value, R squared, adjusted R squared, parameter coefficient, significance, AIC, BIC, standard error, F-statistic, log-likelihood, residual MSE, model MSE, omnibus probability, and JarqueBera probability for all 255 subset combinations of the features. The following subset includes highly significant features based on the *p* values, and the parameters are listed in Table 25, Table 26, Table 27 and Table 28. 

**Figure 11:**
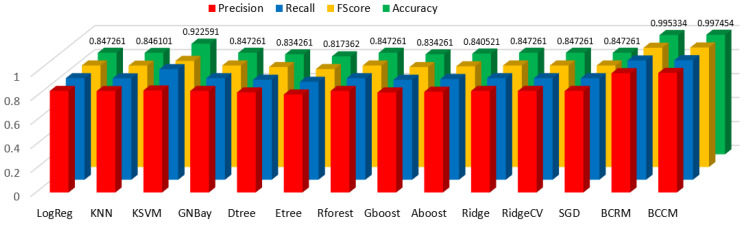
Performance of Proposed BCRM and BCCM with existing classifiers.

Experimental results show that the Gaussian naive Bayes, AdaBoost classifier, and Ridge classifier maintained an accuracy of 89.5% before and after feature scaling for the regressor feature-selection methods. The proposed BCRM was designed with Gaussian naïve Bayes, Ada boost, and Ridge as the estimators and with accuracy optimization using soft blending based on the sum of predicted probabilities of classifiers. The proposed BCRM model showed 99.5% accuracy in predicting hypothyroidism. The implementation results show that the Kernel SVM, KNeighbor, and Ridge classifiers maintained an accuracy of 87.5% before and after feature scaling for the classifier feature-selection methods. The BCCM was created with Kernel SVM, KNeighbor, and Ridge as the estimators with accuracy optimization using soft blending based on the sum of the predicted probabilities of classifiers. The proposed BCCM showed 99.7% accuracy in predicting hypothyroidism. The performance analysis of the proposed BCRM was analyzed with the existing classifiers and the results are shown in Table 29 and Figure 11.

## 5. Conclusions

This paper aimed to predict the existence of hypothyroidism based on an analysis of the features required for classification. The ANOVA test was utilized for the identification of the significant features that predict the target variable. This paper also attempted to apply the regressor and classifier feature-selection algorithms to reduce the dataset with significant features. The dataset was also examined with OLS performance indicators for identification of the best subset of features based on *p* values. The subset feature [‘TSH_measured’, ‘T4U_measured’] has an R squared value of 0.938, which is close to the ideal value. The implementation was carried out with Python in Spyder editor with the Anaconda Navigator IDE. Experimental results show that the Gaussian naive Bayes, AdaBoost classifier, and Ridge classifier maintained an accuracy of 89.5% before and after feature scaling for the regressor feature-selection methods. The MCRM was developed with Gaussian naive Bayes, Ada boost, and Ridge as the estimators, with accuracy optimization using soft blending based on the sum of predicted probabilities of classifiers. The proposed BCRM showed 99.5% accuracy in predicting hypothyroidism. The implementation results show that the Kernel SVM, KNeighbor, and Ridge classifiers maintained an accuracy of 87.5% before and after feature scaling for the classifier feature selection methods. The blunge calibration classifier model was developed with Kernel SVM, KNeighbor, and Ridge as the estimators, with accuracy optimization using soft blending based on the sum of predicted probabilities of classifiers. The proposed blunge calibration classifier model showed 99.7% accuracy in predicting hypothyroidism. As an overview of novelty, the BCCM and BCRM models were built to optimize accuracy with soft blending based on the sum of predicted probabilities of classifiers. The BCRM and BCCM models uniqueness’s are achieved by updating the estimators list with the effective classifiers and regressors that suit the application at runtime. Despite the outstanding performance of the BCRM and BCCM models, it is still difficult for researchers to adjust the model hyper-parameters by combining them with other optimizers and statistical loss functions. 

## Figures and Tables

**Table 1:** Attribute analysis with the ANOVA test.

Features	sum_sq	df	F-Statistic	PR(>F)
Age	4.105	1	55.1339	1.44 × 10^−13^
Sex	2.127	1	28.3421	1.08 × 10^−7^
on_thyroxine	0.920	1	12.1986	0.000485
query_on_thyroxine	0.238	1	3.1494	0.076051
on_antithyroid_medication	0.467	1	6.1798	0.012973
thyroid_surgery	0.024	1	0.3283	0.566654
query_hypothyroid	0.439	1	5.8059	0.016029
query_hyperthyroid	2.556	1	34.1124	5.72 × 10^−9^
pregnant	0.006	1	0.0084	0.926861
sick	0.278	1	3.6821	0.052087
tumor	0.047	1	0.6241	0.429556
lithium	0.013	1	0.1798	0.6715
goitre	2.246	1	29.9307	4.82 × 10^−8^
TSH_measured	117.418	1	3041.1975	0.000045
TSH	0.033	1	0.4446	0.050492
T3_measured	72.038	1	136.1074	5.89 × 10^−248^
T3	0.008	1	0.0111	0.005934
TT4_measured	223.546	1	44,395.61	0.00043
TT4	0.00036	1	0.0047	0.00450
T4U_measured	224.534	1	47,542.78	0.00053
T4U	0.01087	1	0.14349	0.00485
FTI_measured	225.5303	1	51,167.19	0.00034
FTI	0.0036	1	0.0482	0.00049

**Table 2:** Classification metrics before feature scaling.

Classifiers	Precision	Recall	FScore	Accuracy
Logistic regression	0.834285	0.834261	0.834192	0.834261
KNeighbors classifier	0.840521	0.840521	0.840521	0.840521
Kernel SVM classifier	0.851174	0.922591	0.885445	0.922591
Gaussian naive Bayes	0.834285	0.834261	0.834192	0.834261
Decision tree classifier	0.846043	0.846101	0.846064	0.846101
Extra tree classifier	0.834285	0.834261	0.834192	0.834261
Random forest classifier	0.834285	0.834261	0.834192	0.834261
Gradient boosting classifier	0.846043	0.846101	0.846064	0.846101
AdaBoost classifier	0.84363	0.843681	0.84362	0.843681
Ridge classifier	0.834285	0.834261	0.834192	0.834261
Ridge classifierCV	0.834285	0.834261	0.834192	0.834261
SGD classifier	0.834285	0.834261	0.834192	0.834261

**Table 3:** Classification metrics after feature scaling.

Classifiers	Precision	Recall	FScore	Accuracy
Logistic regression	0.847285	0.847261	0.847192	0.847261
KNeighbors classifier	0.846043	0.846101	0.846064	0.846101
Kernel SVM classifier	0.851174	0.922591	0.885445	0.922591
Gaussian baive Bayes	0.847285	0.847261	0.847192	0.847261
Decision tree classifier	0.834285	0.834261	0.834192	0.834261
Extra tree classifier	0.817655	0.817362	0.817477	0.817362
Random forest classifier	0.847285	0.847261	0.847192	0.847261
Gradient boosting classifier	0.834285	0.834261	0.834192	0.834261
AdaBoost classifier	0.840521	0.840521	0.840521	0.840521
Ridge classifier	0.847285	0.847261	0.847192	0.847261
Ridge classifierCV	0.847285	0.847261	0.847192	0.847261
SGD classifier	0.847285	0.847261	0.847192	0.847261

**Table 4:** Regressor feature importance values of each feature before feature scaling.

Index	Classifiers	AdaBoost Regressor	Gradient Boosting Regressor	Extra Trees Regressor	Random Forest Regressor
0.	Age	0.000428341	0.003081406	0.010040952	0.007403365
1.	Sex	0.008622032	4.39 × 10^−5^	0.002254928	0.000698556
2.	on_thyroxine	0	8.62 × 10^−6^	0.000804381	0.000134897
3.	query_on_thyroxine	0	2.19 × 10^−5^	0.000607023	0.00022945
4.	on_antithyroid_medication	0	0	0	0
5.	thyroid_surgery	0	0	1.29 × 10^−5^	0
6.	query_hypothyroid	0	0	0.000137327	0
7.	query_hyperthyroid	0	0.000180298	0.001258513	0.00083488
8.	pregnant	0	1.03 × 10^−20^	0	0
9.	sick	0	0	5.18 × 10^−5^	0
10.	tumor	0	0	6.24 × 10^−7^	0
11.	lithium	0	0	0	0
12.	goitre	0	1.87 × 10^−5^	0.00336961	0.000819246
13.	TSH_measured	0.197501922	0.006246666	0.001243391	0.002119903
14.	TSH	0.077619094	0.001650685	0.002426862	0.002049851
15.	T3_measured	0.017306778	0.000365421	0.001073602	0.00051231
16.	T3	0	0.000614873	0.002119724	0.001266411
17.	TT4_measured	0.004126458	0	5.15 × 10^−5^	0.056711358
18.	TT4	0.053174222	0.013884897	0.010358689	0.01558167
19.	T4U_measured	0	0	0	0.113416878
20.	T4U	0.080253371	0.007295723	0.01125785	0.011729611
21.	FTI_measured	0.505044774	0.957930603	0.943635215	0.774966334
22.	FTI	0.055923007	0.008656368	0.009295157	0.011525281

**Table 5:** Regressor Feature Importance Values of Each Features after Feature Scaling.

Features	AdaBoost	GradientBoosting	ExtraTrees	RandomForest
Age	0.018396825	0.003350051	0.010465358	0.007502654
Sex	0.006847598	4.39 × 10^−5^	0.0022696	0.000651238
on_thyroxine	0	2.43 × 10^−5^	0.000670722	0
query_on_thyroxine	0	2.19 × 10^−5^	0.000453489	0.000176286
on_antithyroid_medication	0	0	0	0
thyroid_surgery	0	0	5.32 × 10^−5^	0
query_hypothyroid	0	1.25 × 10^−6^	0.000134226	0
query_hyperthyroid	0	0.000180298	0.001134248	0.00070627
pregnant	0	0	0	0
sick	0	0	7.78 × 10^−5^	0
tumor	0	0	3.07 × 10^−5^	0
lithium	0	0	0	0
goitre	0	1.87 × 10^−5^	0.003884817	0.000984925
TSH_measured	0.130848738	0.005119183	0.00141153	0.002186828
TSH	0.140185147	0.000508619	0.002227775	0.002164946
T3_measured	0.016544939	0.001884564	0.001113277	0.000384264
T3	0.019546664	1.93 × 10^−5^	0.001694519	0.000764557
TT4_measured	0.002640564	0	0	0.0375457
TT4	0.079190546	0.015118764	0.01054195	0.014953169
T4U_measured	0	0	0	0.170098636
T4U	0.131409566	0.007021985	0.010756814	0.012046173
FTI_measured	0.437162659	0.957930603	0.943635215	0.735679166
FTI	0.017226754	0.008756629	0.009444797	0.014155188

**Table 6:** Classifier feature importance values of each feature before feature scaling.

Features	AdaBoost	Gradient Boosting	Extra Trees	Random Forest
Age	0.3	0.00276237	0.010403875	0.009334997
Sex	0.04	6.31 × 10^−5^	0.003167778	0.001804309
on_thyroxine	0.02	8.98 × 10^−5^	0.002313404	0.001197477
query_on_thyroxine	0.02	9.65 × 10^−6^	0.001441407	0.000280689
on_antithyroid_medication	0	−3.89 × 10^−21^	0.000135951	3.81 × 10^−5^
thyroid_surgery	0	0	0.000158409	9.99 × 10^−5^
query_hypothyroid	0.02	4.13 × 10^−20^	0.000274698	0.000158845
query_hyperthyroid	0.02	0.000142974	0.002732408	0.001816944
pregnant	0	1.67 × 10^−21^	4.77 × 10^−5^	0.000171466
sick	0	0	8.79 × 10^−5^	4.88 × 10^−5^
tumor	0	0	0.000788175	0.000713225
lithium	0	0	0	0
goitre	0	−1.70 × 10^−18^	0.003266947	0.001503002
TSH_measured	0	0.002869076	0.077458279	0.048169136
TSH	0.1	0.001060964	0.003082501	0.046241881
T3_measured	0.04	0.000382842	0.032909932	0.05790805
T3	0.02	5.52 × 10^−6^	0.003573637	0.004400236
TT4_measured	0	2.45 × 10^−6^	0.224022987	0.16221615
TT4	0.16	0.011738893	0.013281768	0.050282253
T4U_measured	0	3.85 × 10^−6^	0.25610996	0.301724198
T4U	0.12	0.008872811	0.013795367	0.045971388
FTI_measured	0.02	0.962267301	0.337492546	0.219637329
FTI	0.12	0.009728344	0.013454301	0.046281594

**Table 7:** Classifier feature importance values of each feature after feature scaling.

Features	AdaBoost	Gradient Boosting	Extra Trees	Random Forest
Age	0.3	0.002453486	0.009509937	0.008279074
Sex	0.04	4.82 × 10^−5^	0.00324165	0.001846886
on_thyroxine	0.02	3.51 × 10^−5^	0.00295889	0.000712379
query_on_thyroxine	0.02	7.33 × 10^−6^	0.000800167	0.000653312
on_antithyroid_medication	0	0	0.000285502	0.000107717
thyroid_surgery	0	0	0.000100357	4.84 × 10^−5^
query_hypothyroid	0.02	−3.89 × 10^−21^	0.000245459	9.70 × 10^−5^
query_hyperthyroid	0.02	0.000138031	0.002982702	0.001717612
pregnant	0	1.07 × 10^−20^	4.60 × 10^−5^	7.20 × 10^−5^
sick	0	0	6.70 × 10^−5^	4.23 × 10^−6^
tumor	0	1.54 × 10^−18^	0.00071482	0.000571423
lithium	0	0	0	0
goitre	0	−1.70 × 10^−18^	0.002505062	0.000921635
TSH_measured	0	0.003078428	0.089211886	0.078112108
TSH	0.1	0.000978036	0.003674423	0.031291888
T3_measured	0.04	0.000575078	0.043737281	0.037562216
T3	0.02	9.39 × 10^−6^	0.003354808	0.009181534
TT4_measured	0	7.58 × 10^−6^	0.1820123	0.205768548
TT4	0.16	0.009939212	0.014136987	0.054038719
T4U_measured	0	0	0.355190855	0.242828144
T4U	0.12	0.008035637	0.013861521	0.035851905
FTI_measured	0.02	0.962709323	0.259146396	0.21946496
FTI	0.12	0.011985167	0.012216028	0.070868256

**Table 8:** Feature importance index of regressor and classifier methods.

Classifiers	Before Feature Scaling	After Feature Scaling
AdaBoost Regressor	13, 14, 18, 20, 21,22	0, 13, 14, 18, 20, 21
GradientBoostingRegressor	0, 13, 18, 20, 21, 22	0, 13, 18, 20, 21, 22
ExtraTrees Regressor	0, 12, 18, 20, 21, 22	0, 12, 18, 20, 21, 22
RandomForest Regressor	17, 18, 19, 20, 21, 22	17, 18, 19, 20, 21, 22
AdaBoost Classifier	0, 1, 14, 18, 20, 22	0, 1, 14, 18, 20, 22
GradientBoosting Classifier	0, 13, 18, 20, 21, 22	0, 13, 18, 20, 21, 22
ExtraTrees Classifier	13, 15, 17, 18, 19, 21	13, 15, 17, 18, 19, 21
RandomForest Classifier	13, 15, 17, 18, 19, 21	13, 15, 17, 18, 19, 21

**Table 9:** AdaBoost regressor metrics before feature scaling.

Classifiers	Precision	Recall	FScore	Accuracy
Logistic regression	0.846043	0.846101	0.846064	0.846101
KNeighbors classifier	0.851174	0.822591	0.885445	0.822591
Kernel SVM classifier	0.847285	0.847261	0.847192	0.847261
Gaussian naive Bayes	0.895285	0.895261	0.895192	0.895261
Decision tree classifier	0.846043	0.846101	0.846064	0.846101
Extra tree classifier	0.851174	0.822591	0.885445	0.822591
Random forest classifier	0.847285	0.847261	0.847192	0.847261
Gradient boosting classifier	0.834285	0.834261	0.834192	0.834261
AdaBoost classifier	0.895285	0.895261	0.895192	0.895261
Ridge classifier	0.895285	0.895261	0.895192	0.895261
Ridge classifierCV	0.846043	0.846101	0.846064	0.846101
SGD classifier	0.834285	0.834261	0.834192	0.834261
Passive aggressive	0.846043	0.846101	0.846064	0.846101
Bagging classifier	0.834285	0.834261	0.834192	0.834261

**Table 10:** AdaBoost regressor metrics after feature scaling.

Classifiers	Precision	Recall	FScore	Accuracy
Logistic regression	0.846043	0.846101	0.846064	0.846101
KNeighbors classifier	0.847285	0.847261	0.847192	0.847261
Kernel SVM classifier	0.834285	0.834261	0.834192	0.834261
Gaussian naive Bayes	0.895285	0.895261	0.895192	0.895261
Decision tree classifier	0.846043	0.846101	0.846064	0.846101
Extra tree classifier	0.851174	0.922591	0.885445	0.882591
Random forest classifier	0.847285	0.847261	0.847192	0.847261
Gradient boosting classifier	0.834285	0.834261	0.834192	0.834261
AdaBoost classifier	0.895285	0.895261	0.895192	0.895261
Ridge classifier	0.895285	0.895261	0.895192	0.895261
Ridge classifierCV	0.834285	0.834261	0.834192	0.834261
SGD classifier	0.846043	0.846101	0.846064	0.846101
Passive aggressive	0.847285	0.847261	0.847192	0.847261
Bagging classifier	0.834285	0.834261	0.834192	0.834261

**Table 11:** Gradient boosting regressor metrics before feature scaling.

Classifiers	Precision	Recall	FScore	Accuracy
Logistic regression	0.834285	0.834261	0.834192	0.834261
KNeighbors classifier	0.846043	0.846101	0.846064	0.846101
Kernel SVM classifier	0.847285	0.847261	0.847192	0.847261
Gaussian naive Bayes	0.895285	0.895261	0.895192	0.895261
Decision tree classifier	0.846043	0.846101	0.846064	0.846101
Extra tree classifier	0.851174	0.822591	0.885445	0.822591
Random forest classifier	0.847285	0.847261	0.847192	0.847261
Gradient boosting classifier	0.834285	0.834261	0.834192	0.834261
AdaBoost classifier	0.895285	0.895261	0.895192	0.895261
Ridge classifier	0.895285	0.895261	0.895192	0.895261
Ridge classifierCV	0.834285	0.834261	0.834192	0.834261
SGD classifier	0.846043	0.846101	0.846064	0.846101
Passive aggressive classifier	0.847285	0.847261	0.847192	0.847261
Bagging classifier	0.834285	0.834261	0.834192	0.834261

**Table 12:** Gradient boosting regressor metrics after feature scaling.

Classifiers	Precision	Recall	FScore	Accuracy
Logistic regression	0.847285	0.847261	0.847192	0.847261
KNeighbors classifier	0.847285	0.847261	0.847192	0.847261
Kernel SVM classifier	0.834285	0.834261	0.834192	0.834261
Gaussian naive Bayes	0.895285	0.895261	0.895192	0.895261
Decision tree	0.846043	0.846101	0.846064	0.846101
Extra tree classifier	0.851174	0.822591	0.885445	0.822591
Random forest classifier	0.847285	0.847261	0.847192	0.847261
GBoosting	0.834285	0.834261	0.834192	0.834261
AdaBoost classifier	0.895285	0.895261	0.895192	0.895261
Ridge classifier	0.895285	0.895261	0.895192	0.895261
Ridge classifierCV	0.834285	0.834261	0.834192	0.834261
SGD classifier	0.847285	0.847261	0.847192	0.847261
Passive Aggressive classifier	0.834285	0.834261	0.834192	0.834261
Bagging classifier	0.834285	0.834261	0.834192	0.834261

**Table 13:** Extra trees regressor metrics before feature scaling.

Classifiers	Precision	Recall	FScore	Accuracy
Logistic regression	0.834285	0.834261	0.834192	0.834261
KNeighbors classifier	0.846043	0.846101	0.846064	0.846101
Kernel SVM classifier	0.851174	0.822591	0.885445	0.822591
Gaussian naive Bayes	0.895285	0.895261	0.895192	0.895261
Decision tree classifier	0.847285	0.847261	0.847192	0.847261
Extra tree classifier	0.847285	0.847261	0.847192	0.847261
Random forest classifier	0.834285	0.834261	0.834192	0.834261
Gradient boosting classifier	0.847285	0.847261	0.847192	0.847261
AdaBoost classifier	0.895285	0.895261	0.895192	0.895261
Ridge classifier	0.895285	0.895261	0.895192	0.895261
Ridge classifierCV	0.847285	0.847261	0.847192	0.847261
SGD classifier	0.834285	0.834261	0.834192	0.834261
Passive Aggressive classifier	0.847285	0.847261	0.847192	0.847261
Bagging classifier	0.834285	0.834261	0.834192	0.834261

**Table 14:** Extra trees regressor metrics after feature scaling.

Classifiers	Precision	Recall	FScore	Accuracy
Logistic regression	0.846043	0.846101	0.846064	0.846101
KNeighbors classifier	0.851174	0.922591	0.885445	0.922591
Kernel SVM classifier	0.847285	0.847261	0.847192	0.847261
Gaussian naïve Bayes	0.895285	0.895261	0.895192	0.895261
Decision tree classifier	0.846043	0.846101	0.846064	0.846101
Extra tree classifier	0.851174	0.922591	0.885445	0.922591
Random Forest classifier	0.847285	0.847261	0.847192	0.847261
Gradient boosting classifier	0.846043	0.846101	0.846064	0.846101
AdaBoost classifier	0.895285	0.895261	0.895192	0.895261
Ridge classifier	0.895285	0.895261	0.895192	0.895261
Ridge classifierCV	0.846043	0.846101	0.846064	0.846101
SGD classifier	0.851174	0.922591	0.885445	0.922591
Passive aggressive classifier	0.847285	0.847261	0.847192	0.847261
Bagging classifier	0.834285	0.834261	0.834192	0.834261

**Table 15:** Random forest regressor metrics before feature scaling.

Classifiers	Precision	Recall	FScore	Accuracy
Logistic regression	0.847285	0.847261	0.847192	0.847261
KNeighbors classifier	0.834285	0.834261	0.834192	0.834261
Kernel SVM classifier	0.851174	0.922591	0.885445	0.922591
Gaussian naive Bayes	0.895285	0.895261	0.895192	0.895261
Decision tree classifier	0.847285	0.847261	0.847192	0.847261
Extra tree Classifier	0.834285	0.834261	0.834192	0.834261
Random forest classifier	0.847285	0.847261	0.847192	0.847261
Gradient boosting classifier	0.834285	0.834261	0.834192	0.834261
AdaBoost classifier	0.895285	0.895261	0.895192	0.895261
Ridge classifier	0.895285	0.895261	0.895192	0.895261
Ridge classifierCV	0.834285	0.834261	0.834192	0.834261
SGD classifier	0.834285	0.834261	0.834192	0.834261
Passive aggressive classifier	0.847285	0.847261	0.847192	0.847261
Bagging classifier	0.834285	0.834261	0.834192	0.834261

**Table 16:** Random forest regressor metrics after feature scaling.

Classifiers	Precision	Recall	FScore	Accuracy
Logistic regression	0.846043	0.846101	0.846064	0.846101
KNeighbors classifier	0.851174	0.822591	0.885445	0.822591
Kernel SVM classifier	0.847285	0.847261	0.847192	0.847261
Gaussian naive Bayes	0.895285	0.895261	0.895192	0.895261
Decision tree classifier	0.846043	0.846101	0.846064	0.846101
Extra tree classifier	0.851174	0.822591	0.885445	0.822591
Random forest classifier	0.847285	0.847261	0.847192	0.847261
Gradient boosting classifier	0.834285	0.834261	0.834192	0.834261
AdaBoost classifier	0.895285	0.895261	0.895192	0.895261
Ridge classifier	0.895285	0.895261	0.895192	0.895261
Ridge classifierCV	0.847285	0.847261	0.847192	0.847261
SGD classifier	0.851174	0.822591	0.885445	0.822591
Passive aggressive classifier	0.847285	0.847261	0.847192	0.847261
Bagging classifier	0.834285	0.834261	0.834192	0.834261

**Table 17:** AdaBoost classifier metrics before feature scaling.

Classifiers	Precision	Recall	FScore	Accuracy
Logistic regression	0.851174	0.822591	0.885445	0.822591
KNeighbors classifier	0.875285	0.877261	0.877192	0.875261
Kernel SVM classifier	0.875285	0.877261	0.877192	0.875261
Gaussian naive Bayes	0.847285	0.847261	0.847192	0.847261
Decision tree classifier	0.851174	0.822591	0.885445	0.822591
Extra tree classifier	0.847285	0.847261	0.847192	0.847261
Random forest classifier	0.834285	0.834261	0.834192	0.834261
Gradient boosting classifier	0.851174	0.822591	0.885445	0.822591
AdaBoost classifier	0.847285	0.847261	0.847192	0.847261
Ridge classifier	0.875285	0.877261	0.877192	0.875261
Ridge classifierCV	0.834285	0.834261	0.834192	0.834261
SGD classifier	0.847285	0.847261	0.847192	0.847261
Passive aggressive classifier	0.834285	0.834261	0.834192	0.834261
Bagging classifier	0.851174	0.822591	0.885445	0.822591

**Table 18:** AdaBoost classifier metrics after feature scaling.

Classifiers	Precision	Recall	FScore	Accuracy
Logistic regression	0.834285	0.834261	0.834192	0.834261
KNeighbors classifier	0.875285	0.877261	0.877192	0.875261
Kernel SVM classifier	0.875285	0.877261	0.877192	0.875261
Gaussian naive Bayes	0.834285	0.834261	0.834192	0.834261
Decision tree classifier	0.834285	0.834261	0.834192	0.834261
Extra tree classifier	0.847285	0.847261	0.847192	0.847261
Random forest classifier	0.834285	0.834261	0.834192	0.834261
Gradient boosting classifier	0.847285	0.847261	0.847192	0.847261
AdaBoost classifier	0.847285	0.847261	0.847192	0.847261
Ridge classifier	0.875285	0.877261	0.877192	0.875261
Ridge classifierCV	0.851174	0.822591	0.885445	0.822591
SGD classifier	0.834285	0.834261	0.834192	0.834261
Passive aggressive classifier	0.847285	0.847261	0.847192	0.847261
Bagging classifier	0.834285	0.834261	0.834192	0.834261

**Table 19:** Gradient boosting classifier metrics before feature scaling.

Classifiers	Precision	Recall	FScore	Accuracy
Logistic regression	0.851174	0.822591	0.885445	0.822591
KNeighbors classifier	0.875285	0.877261	0.877192	0.875261
Kernel SVM classifier	0.875285	0.877261	0.877192	0.875261
Gaussian naive Bayes	0.847285	0.847261	0.847192	0.847261
Decision tree	0.834285	0.834261	0.834192	0.834261
Extra tree	0.847285	0.847261	0.847192	0.847261
Random forest	0.834285	0.834261	0.834192	0.834261
Gradient boosting	0.847285	0.847261	0.847192	0.847261
AdaBoost classifier	0.847285	0.847261	0.847192	0.847261
Ridge classifier	0.875285	0.877261	0.877192	0.875261
Ridge classifierCV	0.851174	0.922591	0.885445	0.922591
SGD classifier	0.847285	0.847261	0.847192	0.847261
Passive aggressive classifier	0.834285	0.834261	0.834192	0.834261
Bagging classifier	0.847285	0.847261	0.847192	0.847261

**Table 20:** Gradient boosting classifier metrics after feature scaling.

Classifiers	Precision	Recall	FScore	Accuracy
Logistic regression	0.834285	0.834261	0.834192	0.834261
KNeighbors classifier	0.875285	0.877261	0.877192	0.875261
Kernel SVM classifier	0.875285	0.877261	0.877192	0.875261
Gaussian naive Bayes	0.834285	0.834261	0.834192	0.834261
Decision tree classifier	0.851174	0.822591	0.885445	0.822591
Extra tree classifier	0.834285	0.834261	0.834192	0.834261
Random forest classifier	0.851174	0.822591	0.885445	0.822591
Gradient boosting classifier	0.834285	0.834261	0.834192	0.834261
AdaBoost classifier	0.834285	0.834261	0.834192	0.834261
Ridge classifier	0.875285	0.877261	0.877192	0.875261
Ridge classifierCV	0.851174	0.922591	0.885445	0.922591
SGD classifier	0.851174	0.822591	0.885445	0.822591
Passive aggressive classifier	0.854541	0.895735	0.87404	0.895735
Bagging classifier	0.834285	0.834261	0.834192	0.834261

**Table 21:** Extra trees classifier metrics before feature scaling.

Classifiers	Precision	Recall	FScore	Accuracy
Logistic regression	0.834285	0.834261	0.834192	0.834261
KNeighbors classifier	0.875285	0.877261	0.877192	0.875261
Kernel SVM classifier	0.875285	0.877261	0.877192	0.875261
Gaussian naive Bayes	0.834285	0.834261	0.834192	0.834261
Decision tree classifier	0.834285	0.834261	0.834192	0.834261
Extra tree classifier	0.851174	0.822591	0.885445	0.822591
Random forest classifier	0.834285	0.834261	0.834192	0.834261
Gradient boosting classifier	0.851174	0.822591	0.885445	0.822591
AdaBoost classifier	0.834285	0.834261	0.834192	0.834261
Ridge classifier	0.875285	0.877261	0.877192	0.875261
Ridge classifierCV	0.834285	0.834261	0.834192	0.834261
SGD classifier	0.851174	0.822591	0.885445	0.822591
Passive aggressive	0.834285	0.834261	0.834192	0.834261
Bagging classifier	0.847285	0.847261	0.847192	0.847261

**Table 22:** Extra trees classifier metrics after feature scaling.

Classifiers	Precision	Recall	FScore	Accuracy
Logistic regression	0.834285	0.834261	0.834192	0.834261
KNeighbors classifier	0.875285	0.877261	0.877192	0.875261
Kernel SVM classifier	0.875285	0.877261	0.877192	0.875261
Gaussian naive Bayes	0.897285	0.897261	0.897192	0.897261
Decision Tree classifier	0.851174	0.822591	0.885445	0.822591
Extra tree classifier	0.834285	0.834261	0.834192	0.834261
Random forest classifier	0.847285	0.847261	0.847192	0.847261
Gradient boosting classifier	0.834285	0.834261	0.834192	0.834261
AdaBoost classifier	0.834285	0.834261	0.834192	0.834261
Ridge classifier	0.875285	0.877261	0.877192	0.875261
Ridge classifierCV	0.851174	0.822591	0.885445	0.822591
SGD classifier	0.834285	0.834261	0.834192	0.834261
Passive aggressive classifier	0.851174	0.822591	0.885445	0.822591
Bagging classifier	0.834285	0.834261	0.834192	0.834261

**Table 23:** Random forest classifier metrics before feature scaling.

Classifiers	Precision	Recall	FScore	Accuracy
Logistic regression	0.851174	0.822591	0.885445	0.822591
KNeighbors classifier	0.875285	0.877261	0.877192	0.875261
Kernel SVM classifier	0.875285	0.877261	0.877192	0.875261
Gaussian naive Bayes	0.897285	0.897261	0.897192	0.897261
Decision tree classifier	0.851174	0.822591	0.885445	0.822591
Extra tree classifier	0.834285	0.834261	0.834192	0.834261
Random forest classifier	0.847285	0.847261	0.847192	0.847261
Gradient boosting classifier	0.831174	0.832591	0.835445	0.832591
AdaBoost classifier	0.834285	0.834261	0.834192	0.834261
Ridge classifier	0.875285	0.877261	0.877192	0.875261
Ridge classifierCV	0.834285	0.834261	0.834192	0.834261
SGD classifier	0.847285	0.847261	0.847192	0.847261
Passive aggressive classifier	0.831174	0.832591	0.835445	0.832591
Bagging classifier	0.847285	0.847261	0.847192	0.847261

**Table 24:** Random forest classifier metrics after feature scaling.

Classifiers	Precision	Recall	FScore	Accuracy
Logistic regression	0.847285	0.847261	0.847192	0.847261
KNeighbors classifier	0.875285	0.877261	0.877192	0.875261
Kernel SVM classifier	0.875285	0.877261	0.877192	0.875261
Gaussian naive Bayes	0.834285	0.834261	0.834192	0.834261
Decision tree classifier	0.851174	0.822591	0.885445	0.822591
Extra tree classifier	0.834285	0.834261	0.834192	0.834261
Random forest classifier	0.847285	0.847261	0.847192	0.847261
Gradient boosting classifier	0.831174	0.832591	0.835445	0.832591
AdaBoost classifier	0.834285	0.834261	0.834192	0.834261
Ridge classifier	0.875285	0.877261	0.877192	0.875261
Ridge classifierCV	0.851174	0.822591	0.885445	0.822591
SGD classifier	0.834285	0.834261	0.834192	0.834261
Passive aggressive	0.847285	0.847261	0.847192	0.847261
Bagging classifier	0.831174	0.832591	0.835445	0.832591

**Table 25:** OLS features of the significant subset attributes of the hypothyroidism dataset.

S.No	Attributes	R Squared	Adjusted R Squared	Parameter Coefficient
1.	[‘TSH_measured’]	0.490342	0.490181	[−0.1926723]
2.	[‘TSH_measured’ ‘TSH’ ‘TT4_measured’]	0.934966	0.934904	[−0.01378328 0.00334404 −0.25622659]
3.	[‘TSH_measured’ ‘TSH’ ‘T4U_measured’]	0.939086	0.939028	[−0.01372514 0.00334439 −0.25687507]
4.	[‘TSH_measured’ ‘T3_measured’]	0.508019	0.507707	[−0.16245081 −0.04745117]
5.	[‘TSH_measured’ ‘T3_measured’ ‘T3’]	0.508773	0.508306	[−0.16300138 −0.04710052 −0.00756629]
6.	[‘TSH_measured’ ‘T3’]	0.491378	0.491056	[−0.19305575 −0.00886611]
7.	[‘TSH_measured’ ‘T3’ ‘T4U’]	0.492164	0.491682	[−0.19327524 −0.01212442 0.00837232]
8.	[‘TSH_measured’ ‘TT4_measured’]	0.934818	0.934777	[−0.01378472 −0.25622453]
9.	[‘TSH_measured’ ‘T4U_measured’]	0.938938	0.9389	[−0.01372657 −0.25687302]
10.	[‘TSH’ ‘T3_measured’ ‘TT4_measured’]	0.934199	0.934136	[0.00338423 −0.00749316 −0.26174326]
11.	[‘TSH’ ‘T3_measured’ ‘T4U_measured’]	0.938323	0.938265	[0.0033845 −0.00747897 −0.26234687]
12.	[‘TSH’ ‘TT4_measured’]	0.93368	0.933638	[0.00334707 −0.26584963]
13.	[‘TSH’ ‘T4U_measured’]	0.937806	0.937766	[0.00334741 −0.26643643]
14.	[‘T3_measured’]	0.300835	0.300614	[−0.15091554]
15.	[‘T3_measured’ ‘TT4_measured’]	0.934047	0.934006	[−0.00746918 −0.26175534]
16.	[‘T3_measured’ ‘TT4’]	0.302135	0.301694	[−0.15159006 −0.00994458]
17.	[‘T3_measured’ ‘T4U_measured’]	0.938172	0.938133	[−0.00745503 −0.26235889]
18.	[‘TT4_measured’]	0.933532	0.933511	[−0.26584857]
19.	[‘T4U_measured’]	0.937658	0.937638	[−0.26643537]

**Table 26:** OLS features of the significant subset attributes of the hypothyroid dataset.

S.No	Attributes	*p* Values	AIC	BIC
1.	[‘TSH_measured’]	[0.]	−1315.02	−1302.9
2.	[‘TSH_measured’ ‘TSH’ ‘TT4_measured’]	[3.69199042 × 10^−15^ 7.43156772 × 10^−3^ 0.00000000]	−7823.1	−7798.86
3.	[‘TSH_measured’ ‘TSH’ ‘T4U_measured’]	[5.19971275 × 10^−16^ 5.67318032 × 10^−3^ 0.00000000]	−8030.12	−8005.88
4.	[‘TSH_measured’ ‘T3_measured’]	[1.81373646 × 10^−243^ 4.53106996 × 10^−26^]	−1424.67	−1406.49
5.	[‘TSH_measured’ ‘T3_measured’ ‘T3’]	[1.93109194 × 10^−244^ 1.02662783 × 10^−25^ 2.77571221 × 10^−2^]	−1427.52	−1403.28
6.	[‘TSH_measured’ ‘T3’]	[0. 0.01121299]	−1319.46	−1301.28
7.	[‘TSH_measured’ ‘T3’ ‘T4U’]	[0. 0.00139307 0.02711176]	−1322.35	−1298.11
8.	[‘TSH_measured’ ‘TT4_measured’]	[3.89722887 × 10^−15^ 0.00000000]	−7817.92	−7799.74
9.	[‘TSH_measured’ ‘T4U_measured’]	[5.53480256 × 10^−16^ 0.00000000]	−8024.46	−8006.28
10.	[‘TSH’ ‘T3_measured’ ‘TT4_measured’]	[7.07863933 × 10^−3^ 6.32943158 × 10^−7^ 0.00000000]	−7786	−7761.76
11.	[‘TSH’ ‘T3_measured’ ‘T4U_measured’]	[5.40529071 × 10^−3^ 2.75925083 × 10^−7^ 0.00000000]	−7990.75	−7966.52
12.	[‘TSH’ ‘TT4_measured’]	[0.00796318 0. ]	−7763.15	−7744.97
13.	[‘TSH’ ‘T4U_measured’]	[0.00613635 0. ]	−7966.31	−7948.13
14.	[‘T3_measured’]	[5.89765862 × 10^−248^]	−315.046	−302.927
15.	[‘T3_measured’ ‘TT4_measured’]	[7.04200951 × 10^−7^ 0.00000000]	−7780.73	−7762.56
16.	[‘T3_measured’ ‘TT4’]	[3.72603592 × 10^−249^ 1.53025228 × 10^−2^]	−318.934	−300.756
17.	[‘T3_measured’ ‘T4U_measured’]	[3.09674931 × 10^−7^ 0.00000000]	−7985	−7966.83
18.	[‘TT4_measured’]	[0.]	−7758.1	−7745.98
19.	[‘T4U_measured’]	[0.]	−7960.79	−7948.67

**Table 27:** OLS features of the significant subset attributes of the hypothyroid dataset.

S.No	Attributes	StandardError	FStatistic	Likelihood
1.	[‘TSH_measured’]	[0.00349379]	3041.198	659.5089
2.	[‘TSH_measured’ ‘TSH’ ‘TT4_measured’]	[0.00174378 0.00124843 0.00174378]	15,138.54	3915.549
3.	[‘TSH_measured’ ‘TSH’ ‘T4U_measured’]	[0.00168412 0.00120824 0.00168412]	16,233.73	4019.059
4.	[‘TSH_measured’ ‘T3_measured’]	[0.00445323 0.00445323]	1631.505	715.3352
5.	[‘TSH_measured’ ‘T3_measured’ ‘T3’]	[0.00445754 0.00445337 0.00343653]	1090.61	717.7602
6.	[‘TSH_measured’ ‘T3’]	[0.00349406 0.00349406]	1526.435	662.7281
7.	[‘TSH_measured’ ‘T3’ ‘T4U’]	[0.00349332 0.00379016 0.00378678]	1020.505	665.1734
8.	[‘TSH_measured’ ‘TT4_measured’]	[0.00174548 0.00174548]	22,659.94	3911.961
9.	[‘TSH_measured’ ‘T4U_measured’]	[0.0016859 0.0016859]	24,295.55	4015.228
10.	[‘TSH’ ‘T3_measured’ ‘TT4_measured’]	[0.0012558 0.00150131 0.00150129]	14,949.73	3896.998
11.	[‘TSH’ ‘T3_measured’ ‘T4U_measured’]	[0.0012158 0.00145213 0.00145211]	16,019.93	3999.377
12.	[‘TSH’ ‘TT4_measured’]	[0.00126052 0.00126052]	22,243.82	3884.576
13.	[‘TSH’ ‘T4U_measured’]	[0.00122068 0.00122068]	23,824.18	3986.153
14.	[‘T3_measured’]	[0.00409211]	1360.107	159.5229
15.	[‘T3_measured’ ‘TT4_measured’]	[0.00150277 0.00150277]	22,376.61	3893.367
16.	[‘T3_measured’ ‘TT4’]	[0.00409839 0.00409839]	684.0491	162.4668
17.	[‘T3_measured’ ‘T4U_measured’]	[0.00145365 0.00145365]	23,974.81	3995.502
18.	[‘TT4_measured’]	[0.00126172]	44,395.61	3881.051
19.	[‘T4U_measured’]	[0.00122194]	47,542.78	3982.394

**Table 28:** OLS features of the significant subset attributes of the hypothyroid dataset.

S.No	Attributes	Residual MSE	Model MSE	Omnibus Probability	JarqueBera Probability
1.	[‘TSH_measured’]	0.038609	0.075732	2.61 × 10^−76^	0
2.	[‘TSH_measured’ ‘TSH’ ‘TT4_measured’]	0.00493	0.075732	0	0
3.	[‘TSH_measured’ ‘TSH’ ‘T4U_measured’]	0.004617	0.075732	0	0
4.	[‘TSH_measured’ ‘T3_measured’]	0.037282	0.075732	2.49 × 10^−75^	0
5.	[‘TSH_measured’ ‘T3_measured’ ‘T3’]	0.037237	0.075732	1.90 × 10^−75^	0
6.	[‘TSH_measured’ ‘T3’]	0.038543	0.075732	4.64 × 10^−76^	0
7.	[‘TSH_measured’ ‘T3’ ‘T4U’]	0.038496	0.075732	7.40 × 10^−76^	0
8.	[‘TSH_measured’ ‘TT4_measured’]	0.004939	0.075732	0	0
9.	[‘TSH_measured’ ‘T4U_measured’]	0.004627	0.075732	0	0
10.	[‘TSH’ ‘T3_measured’ ‘TT4_measured’]	0.004988	0.075732	0	0
11.	[‘TSH’ ‘T3_measured’ ‘T4U_measured’]	0.004675	0.075732	0	0
12.	[‘TSH’ ‘TT4_measured’]	0.005026	0.075732	0	0
13.	[‘TSH’ ‘T4U_measured’]	0.004713	0.075732	0	0
14.	[‘T3_measured’]	0.052966	0.075732	0	0
15.	[‘T3_measured’ ‘TT4_measured’]	0.004998	0.075732	0	0
16.	[‘T3_measured’ ‘TT4’]	0.052884	0.075732	0	0
17.	[‘T3_measured’ ‘T4U_measured’]	0.004685	0.075732	0	0
18.	[‘TT4_measured’]	0.005035	0.075732	0	0
19.	[‘T4U_measured’]	0.004723	0.075732	0	0

**Table 29:** Performance analysis of proposed BCRM and BCCM with existing classifiers.

Classifiers	Precision	Recall	FScore	Accuracy
Logistic regression	0.847285	0.847261	0.847192	0.847261
KNeighbors classifier	0.846043	0.846101	0.846064	0.846101
Kernel SVM classifier	0.851174	0.922591	0.885445	0.922591
Gaussian naive Bayes	0.847285	0.847261	0.847192	0.847261
Decision tree classifier	0.834285	0.834261	0.834192	0.834261
Extra tree classifier	0.817655	0.817362	0.817477	0.817362
Random forest classifier	0.847285	0.847261	0.847192	0.847261
Gradient boosting	0.834285	0.834261	0.834192	0.834261
AdaBoost classifier	0.840521	0.840521	0.840521	0.840521
Ridge classifier	0.847285	0.847261	0.847192	0.847261
Ridge classifierCV	0.847285	0.847261	0.847192	0.847261
SGD classifier	0.847285	0.847261	0.847192	0.847261
Proposed BCRM	0.995234	0.995224	0.995334	0.995334
Proposed BCCM	0.997432	0.997422	0.997432	0.997454

## Data Availability

Not applicable.
